# Potentiation of endocannabinoids and other lipid amides prevents hyperalgesia and inflammation in a pre-clinical model of migraine

**DOI:** 10.1186/s10194-022-01449-1

**Published:** 2022-07-07

**Authors:** Rosaria Greco, Chiara Demartini, Anna Maria Zanaboni, Miriam Francavilla, Angelo Reggiani, Natalia Realini, Rita Scarpelli, Daniele Piomelli, Cristina Tassorelli

**Affiliations:** 1 Unit of Translational Neurovascular Research, IRCCS Mondino Foundation, Pavia, Italy; 2 Department of Brain and Behavioral Sciences, University of Pavia, Pavia, Italy; 3 Drug Discovery and Development (D3)-Validation, Fondazione Istituto Italiano di Tecnologia, Genoa, Italy; 4 Department of Anatomy and Neurobiology, University of California, Irvine, CA USA

**Keywords:** FAAH inhibitors, NTG, Trigeminal hyperalgesia, Lipid-derived mediators

## Abstract

Targeting fatty acid amide hydrolase (FAAH) is a promising therapeutic strategy to combat certain forms of pain, including migraine headache. FAAH inhibitors, such as the *O*-biphenyl-3-yl carbamate URB597, have been shown to produce anti-hyperalgesic effects in animal models of migraine. The objective of this study was to investigate the behavioral and biochemical effects of compounds ARN14633 and ARN14280, two URB597 analogs with improved solubility and bioavailability, in a migraine-specific rat model in which trigeminal hyperalgesia is induced by nitroglycerin (NTG) administration. ARN14633 (1 mg/kg, i.p.) and ARN14280 (3 mg/kg, i.p.) were administered to adult male Sprague-Dawley rats 3 hours after NTG injection. One hour after the administration of either compound, rats were subjected to the orofacial formalin test. ARN14633 and ARN14280 attenuated NTG-induced nocifensive behavior and reduced transcription of genes encoding neuronal nitric oxide synthase, pain mediators peptides (calcitonin gene-related peptide, substance P) and pro-inflammatory cytokines (tumor necrosis factor-alpha, interleukin-1beta and 6) in the trigeminal ganglion, cervical spinal cord and medulla. Finally, both compounds strongly elevated levels of endocannabinoids and/or other FAAH substrates in cervical spinal cord and medulla, and, to a lesser extent, in the trigeminal ganglia. The results indicate that the novel global FAAH inhibitors ARN14633 and ARN14280 elicit significant anti-hyperalgesic effects in a migraine-specific animal model and inhibit the associated peptidergic-inflammatory response. Although the precise mechanism underlying these effects remains to be elucidated, our results support further investigational studies of FAAH blockade as a potential therapeutic strategy to treat migraine conditions.

**Supplementary Information:**

The online version contains supplementary material available at 10.1186/s10194-022-01449-1.

## Introduction

Preclinical evidence suggests fatty acid amide hydrolase (FAAH) enzyme  as a potential therapeutic target for migraine headache [1, 2]. FAAH is expressed in the central nervous system as well as in most peripheral organ systems [3–5], where it deactivates a functionally heterogenous family of bioactive lipid amides involved in the regulation of pain, inflammation and other physiological processes. In addition to the endocannabinoid arachidonoylethanolamide (AEA, anandamide), FAAH catalyzes the hydrolysis of palmitoylethanolamide (PEA) and oleoylethanolamide (OEA), whose biological actions are primarily mediated by nuclear peroxisome proliferator-activated receptor-α (PPAR-α) [6–8]. The first potent, systemically active and selective FAAH inhibitor to be disclosed was the *O*-biphenyl-3-yl carbamate URB597 [9, 10]. This agent displays marked anxiolytic-like [9] and antidepressant-like properties in rodent models [11, 12] and attenuates addictive-like behaviors in both rodents and non-human primates [13, 14]. Most of these effects can be attributed to enhanced activation of the cannabinoid-1 (CB_1_) and CB_2_ receptors by non-metabolized AEA [9, 11, 15, 16], though a contribution of OEA/PEA-dependent activation of PPAR-α was also described [17, 18].

**Fig. 1:**
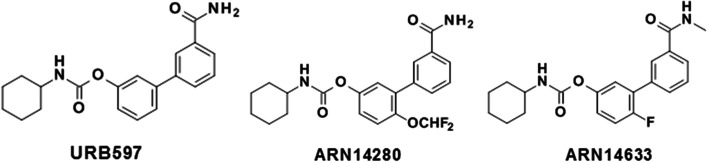
Chemical structures of URB597, ARN14280 and ARN14633

URB597 alleviates sensory abnormalities in several animal models of acute and chronic pain, but not in others. For example, repeated administration of the compound reduced nociceptive responses in the mouse chronic constriction injury model of neuropathic pain [19], but not in the carrageenan model of inflammatory pain [20]. Lack of antinociceptive efficacy was also reported after subchronic URB597 treatment in the spinal cord injury model of neuropathic pain [21]. Similarly, we recently showed that a single URB597 administration effectively countered facial hypersensitivity induced by nitroglycerin (NTG), a rat model of migraine, when administered prior to but not after NTG [2]. In the present study we investigated the effects of two URB597 analogs with improved solubility and bioavailability – ARN14633 ([4-fluoro-3-[3-(methylcarbamoyl)phenyl]phenyl] N-cyclohexylcarbamate) and ARN14280 ([3-(3-carbamoylphenyl)-4-(difluoromethoxy)phenyl] N-cyclohexylcarbamate) (Fig. 1) – in the NTG model of migraine. We focused on anti-hyperalgesic activity in the trigeminal area and on inhibition of the peptidergic and inflammatory response associated with NTG administration.

## Methods

### Synthesis and chemical optimization of FAAH inhibitors

ARN14280 ([3-(3-carbamoylphenyl)-4-(difluoromethoxy)phenyl] *N*-cyclohexylcarbamate) and ARN14633 ([4-fluoro-3-[3-(methylcarbamoyl) phenyl]phenyl] *N*-cyclohexylcarbamate) were prepared according to synthetic procedures as described in the patent application WO2015157313. Briefly, ARN14280 was synthetized in a four-step procedure, starting from commercially available 4-benzyloxy-2-bromo-phenol, through a difluoromethylation reaction (diethyl bromodifluoromethylphosphonate, KOH, CH_3_CN, − 10 °C to room temperature, 2 h), followed by Suzuki cross-coupling reaction (3-carbamoylbenzeneboronic acid, Pd (OAc)_2_, K_3_CO_3_, EGME/H_2_O, 50 °C, 20 min, 95% over 2 steps), a Pd/C catalyzed hydrogenative deprotection (10% Pd/C, cyclohexene, 2-MeTHF, reflux, 2 h) and a carbamoylation reaction (cyclohexyl isocyanate, Et_3_N, CH_3_CN, rt., 16 h, 71% over 2 steps). ARN14633 was synthetized in a two-step procedure starting from the commercially available 3-bromo-4-fluoro-phenol, which was converted into 3-(2-fluoro-5-hydroxy-phenyl)-*N*-methyl-benzamide through a Suzuki cross-coupling reaction (Pd(OAc)_2_, S-Phos, K_3_PO_4_, THF/H_2_O, 50 °C, 2 h, 88%), followed by carbamoylation (cyclohexyl isocyanate, DMAP, DCM, rt., 16 h, 72%).

The chemical optimization strategy was mainly focused on designing and synthetizing a set of new analogs, bearing a variety of structural modifications on the three main regions A, B, and C of the URB597 scaffold (Fig. 1S, supplementary material), with the aim of optimizing the pharmacokinetic profile of this class of *O*-biphenyl-3-yl carbamate FAAH inhibitors. Our approach consisted of evaluating the effects of the insertion of different aliphatic *N*-linked groups (left side region) and different substituents on the proximal and the distal phenyl rings (central region and right side region, respectively) of URB597, on inhibitory activity and drug-like properties (Fig. 1S, supplementary material). ARN14633 bears a fluorine atom at the *para*- position on the proximal phenyl ring and a methyl group on the primary carboxamide functionality at the 3′-position of the distal phenyl ring, while ARN14280 is derived by the introduction of a di-fluoromethoxy group at the *para* -position on the proximal phenyl ring of URB597. The detailed medicinal chemistry exploration will be the subject of a separate publication. ARN14633 and ARN14280 are potent FAAH inhibitors (r-FAAH IC50 = 1.4 ± 0.3 and 1.5 ± 0.3 nM, respectively) with improved kinetic solubility and oral bioavailability in rats (kinetic solubility = 28 and 44 μM; oral bioavailability F = 87% and 35%, respectively) compared to the parent URB597 (kinetic solubility = 1 μM; oral bioavailability, F = 5%).

### Animals

A total of 96 adult male Sprague-Dawley rats, weighing 250–270 g, were randomly allocated to experimental groups as reported in Table 1. The IASP’s guidelines for pain research in animals were followed [22]. All procedures were conducted in accordance with the European Convention for Care and Use of Laboratory Animals and the experimental protocols were approved by the Italian Ministry of Health (1032/2015-PR; 1019/2016-PR; 1239/2015-PR).

**Table 1:** Experimental groups and number (N) of animals used in different sets, with different procedures: orofacial formalin test (OFT); gene expression analysis (mRNA); AEA, PEA, OEA and 2-AG level analysis in medulla, cervical spinal cord, and trigeminal ganglion

Treatment	Group A OFT + mRNA (N)	Group B Lipids (N)
**CT (NTG vehicle + inhibitors vehicle)**	9	6
**ARN14633 (ARN14633 + NTG vehicle)**	9	6
**ARN14280 (ARN14280 + NTG vehicle)**	9	6
**NTG (NTG + inhibitors vehicle)**	9	6
**NTG + ARN14633**	9	6
**NTG + ARN14280**	9	6
**NTG + URB597**	6 (only OFT)	–

### Experimental design

**Fig. 2:**
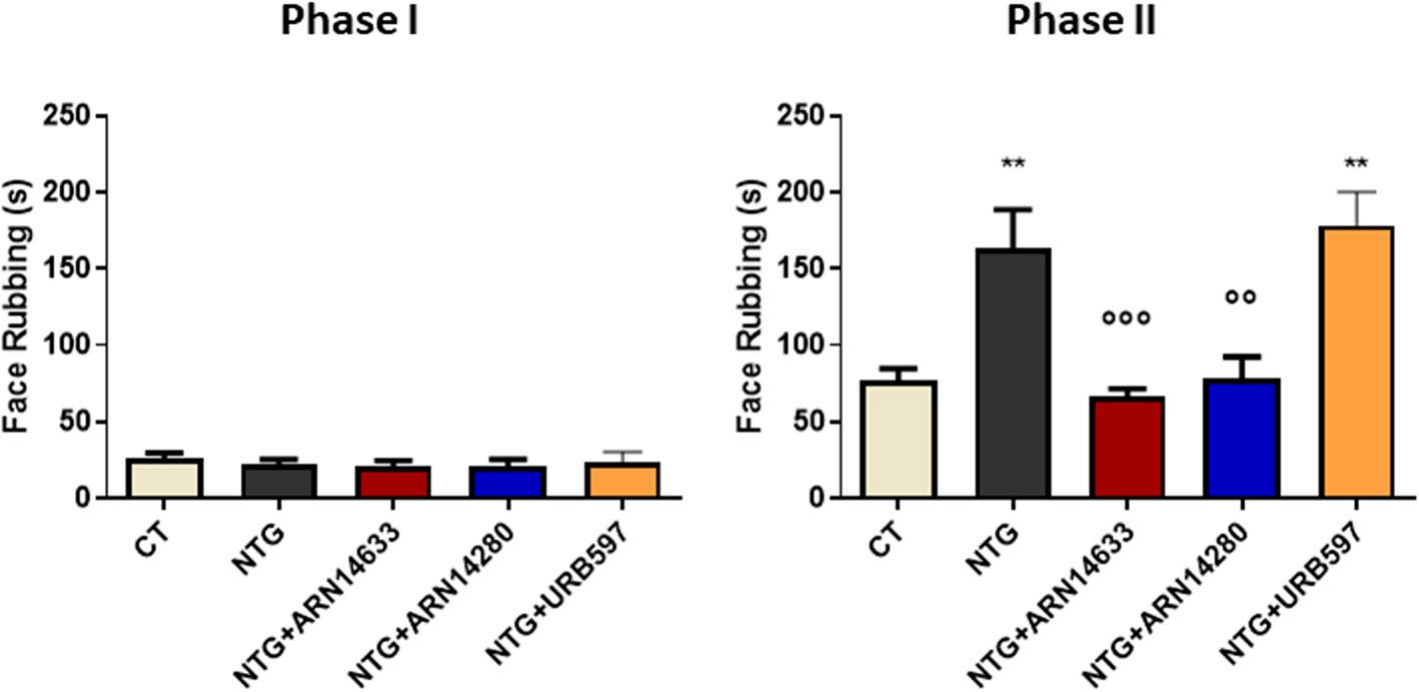
Time spent in face rubbing behavior (seconds) during Phase I (0–3 minutes) and II (12–45 minutes) of the orofacial formalin test in rats treated with NTG vehicle (CT), NTG, NTG + ARN14663 or ARN14280 or URB597 (*n* = 6–9 per group). Data are reported as mean ± SEM. One-way ANOVA followed by Tukey’s multiple comparisons test: **p < 0.01 vs CT; °°*p* < 0.01 and °°°*p* < 0.001 vs NTG and NTG + URB597

NTG (Bioindustria L.I.M. Novi Ligure (AL), Italy) was prepared as previously described [23]; animals received intraperitoneal (i.p.) injection of NTG (10 mg/Kg) or its vehicle (16% propylene glycol, 6% alcohol and saline) 4 hours before testing and/or ex vivo analysis [2] (Table 1 and Fig. 2S, supplementary material). ARN14633 and ARN14280 were dissolved in 10% PEG200, 10% Tween 80 and saline [24], and administered i.p. at the dose of 1 and 3 mg/kg respectively. The dose was selected based on previous studies [25] and on the overall physicochemical and metabolic properties of the *O*-biphenyl-3-yl carbamate URB597, of which they are close analogs. The structure-activity and structure-property relationship studies that lead to the identification of these compounds will be the subject of a separate publication.

URB597 (3′-carbamoyl-biphenyl-3-yl-cy-clohexylcarbamate, Cayman Chemical) was dissolved in the same vehicle and was injected i.p. at a dose of 2 mg/kg [2, 26].

Rats were given NTG or its vehicle at baseline and, 3 hours later, were treated with ARN14633, ARN14280, URB597, or their vehicle. One hour later (i.e., 4 hours from NTG/vehicle administration), a group of rats (group A) underwent the orofacial formalin test for behavioral assessment and were sacrificed at the end of the test to evaluate gene expression in medulla, cervical spinal cord (CSC) and trigeminal ganglion (TG). The second group (group B) was instead sacrificed (4 hours from NTG/vehicle administration) without being exposed to the orofacial formalin test to measure AEA, PEA, OEA, and 2-arachidonylglycerol (2-AG) in medulla, CSC and TG (Table 1 and Fig. 2S, supplementary material).

### Orofacial formalin test

The procedures applied for the behavioral test have been described elsewhere [2, 23, 27]. Briefly, after acclimatization (20 min) to the test chamber, rats were given subcutaneous injections of formalin (1.5%, 50 μL) into the right upper lip. Face rubbing was measured by a researcher blind to treatments counting the seconds the animal spent grooming the injected area with the ipsilateral forepaw or hind paw 0–3 min (Phase I) and 12–45 min (Phase II) after formalin injection.

### Gene expression analysis

At the end of the behavioral test, rats were euthanized with a lethal dose of anesthetic for tissue sample collection. The medulla (bregma, − 13.30 to − 14.60 mm) was dissected out in toto, while we collected CSC (C1-C2) and TG ipsilateral to the formalin injection site. Samples were rinsed in ice-cold sterile 0.9% saline, placed in cryogenic tubes, and immediately frozen in liquid nitrogen. They were subsequently kept at − 80 °C until rt-PCR processing. mRNA levels of genes encoding for c-Fos, CGRP, substance P (SP), nNOS, Tumor Necrosis Factor alpha (TNF-alpha), Interleukin 6 (IL-6) and IL-1beta (primer sequences are reported in Table 2) were measured by rt-PCR as previously reported [2, 27, 28]. Glyceraldehyde 3-phosphate dehydrogenase (GAPDH), whose expression remained constant in all experimental groups, was used for normalization. All samples were assayed in triplicate and gene expression levels were calculated according to 2^−∆∆Ct^ = 2^− (∆Ct gene − ∆Ct housekeeping gene)^ formula by using Ct (cycle threshold) values.

**Table 2:** Primer sequences obtained from the AutoPrime software (http://www.autoprime.de/AutoPrimeWeb) and validated on BLAST

Gene	Forward primer	Reverse primer
**GAPDH**	AACCTGCCAAGTATGATGAC	GGAGTTGCTGTTGAAGTCA
**c-fos**	TACGCTCCAAGCGGAGAC	TTTCCTTCTCTTTCAGTAGATTGG
**nNOS**	CCGGCTACACTTCTCCTCAC	CACGAAGCAGGGGACTACAT
**CGRP**	CAGTCTCAGCTCCAAGTCATC	TTCCAAGGTTGACCTCAAAG
**SP**	GCTCTTTATGGGCATGGTC	GGGTTTATTTACGCCTTCTTTC
**IL-1beta**	CTTCCTTGTGCAAGTGTCTG	CAGGTCATTCTCCTCACTGTC
**IL-6**	TTCTCTCCGCAAGAGACTTC	GGTCTGTTGTGGGTGGTATC
**TNF-alpha**	CCTCACACTCAGATCATCTTCTC	CGCTTGGTGGTTTGCTAC

### AEA, PEA, OEA and 2-AG levels

For the evaluation of lipid concentrations in central and peripheral areas related to trigeminal pain, rats were given ARN14633, ARN14280 or vehicle and were sacrificed by decapitation after exposure to carbon dioxide [29]. Since several factors may influence the concentrations of endogenous substances measured in post-mortem tissue samples, medulla (bregma, −13.30 to −14.60 mm), CSC (C1-C2) and TGs were quickly dissected out in toto and snap-frozen in liquid N_2_. For analyses, frozen tissue samples were homogenized and lipids were extracted using a Bligh-Dyer procedure, modified as previously described [30]. Briefly, tissues were weighed, transferred to glass vials, and homogenized in cold methanol (2 mL) containing PEA-*d*_*4*_, OEA-*d*_*4*_, AEA-*d*_*4*_ and 2-AG-*d*_*8*_ as internal standards [30]. Lipids were extracted with chloroform (2 mL) and washed with LC/MS-grade water (1 mL). After centrifugation for 15 min at 2850  × *g* and 4 °C, the organic phases were collected and transferred to a new set of glass vials. To increase extraction efficiency, the aqueous fractions were extracted again with chloroform (1 mL) and the centrifugation step was repeated. Both organic phases were pooled and dried under N_2_. Lipids were reconstituted in chloroform (2 mL) and the organic extracts were fractionated by Silica Gel G column chromatography (60-Å 230–400 mesh; Sigma-Aldrich, Milan, Italy). PEA, OEA, AEA and 2-AG levels were eluted from the silica column with 2 mL of chloroform/methanol (9:1, v/v) and then with 2 mL of chloroform/methanol (8:2, v/v). Both eluates were recovered in the same vial. The solvent was evaporated under N_2_ and lipids were reconstituted in methanol/chloroform (50 μL; 9:1, v/v) and transferred to glass vials for LC/MS analyses. PEA, OEA, AEA and 2-AG levels were measured using a Xevo TQ UPLC-MS/MS system equipped with a reversed-phase BEH C18 column (2.1  ×  50 mm, 1.7 μm particle size) (Waters, Milford, USA). The mobile phase consisted of 0.1% formic acid in water as solvent A and 0.1% formic acid in acetonitrile as solvent B. A linear gradient was used: 0.0–0.5 min 20% B; 0.5–2.5 min 20 to 100% B; and 2.5–3.0 min maintained at 100% B. The column was reconditioned to 20% B for 1 min. Analysis time was 4 min and the injection volume was 5 μL.

### Statistical analysis

To determine the minimal sample size needed to achieve the experiments, we considered as the primary outcome the nociceptive response in Phase II of the orofacial formalin test (face rubbing time). An a priori power analysis was conducted to obtain a statistical power of 0.80 at an alpha level of 0.05 (GPower 3.1). We hypothesized a difference in total face rubbing time between NTG-treated rats (mean 170  ± 14) and those injected with NTG + inhibitors of at least 25 s (mean 145  ± 18) and thus, we estimated a sample size of 6 rats in each experimental group with an effect size of 1.55. However, due to the intergroup variability seen in the orofacial formalin test, we used a maximum of 9 rats *per* group.

For nociceptive responses, gene expression and lipid levels, the statistical differences between groups were determined using the one-way ANOVA followed by post hoc Tukey’s Multiple Comparison Test; a probability level of less than 5% was regarded as significant.

## Results

### Anti-hyperalgesic effects of FAAH inhibitors in the orofacial formalin test

**Fig. 3:**
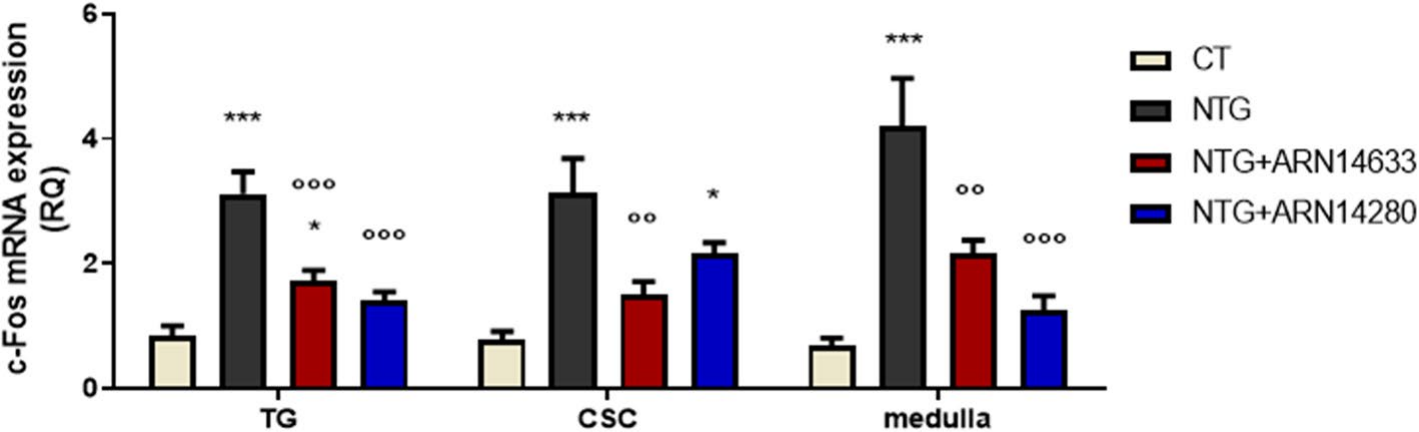
C-fos mRNA levels (expressed as relative quantification, RQ) in trigeminal ganglion (TG) and cervical spinal cord (CSC) ipsilateral to formalin injection and medulla in toto of animals treated with NTG vehicle (CT), NTG, NTG + ARN14663 and NTG + ARN14280 (n. 9 per group). Data are expressed as mean ± SEM; one-way ANOVA followed by Tukey’s multiple comparisons test; *p < 0.05 and ***p < 0.001 vs CT; °°*p* < 0.01 and °°°*p* < 0.001 vs NTG

Administration of NTG did not enhance Phase I nocifensive behavior (first 0–3 min of face rubbing) in formalin-injected rats (Fig. 2). Administration of the FAAH inhibitors was also without effect (Fig. 2). As previously reported [2, 23], NTG caused a significant increase in Phase II behavior (12–45 min of face rubbing) relative to control vehicle-treated animals (CT). Both ARN14280 (1 mg/kg) and ARN14633 (3 mg/kg) significantly attenuated the response to NTG whereas, in agreement with previous work [2], URB597 had no such effect (Fig. 2). ARN14280 and ARN14633 did not show any effect on either phase of the test when administered to rats that were not pre-treated with NTG (Fig. 3S, supplementary material).

### Inhibition of gene expression

**Fig. 4:**
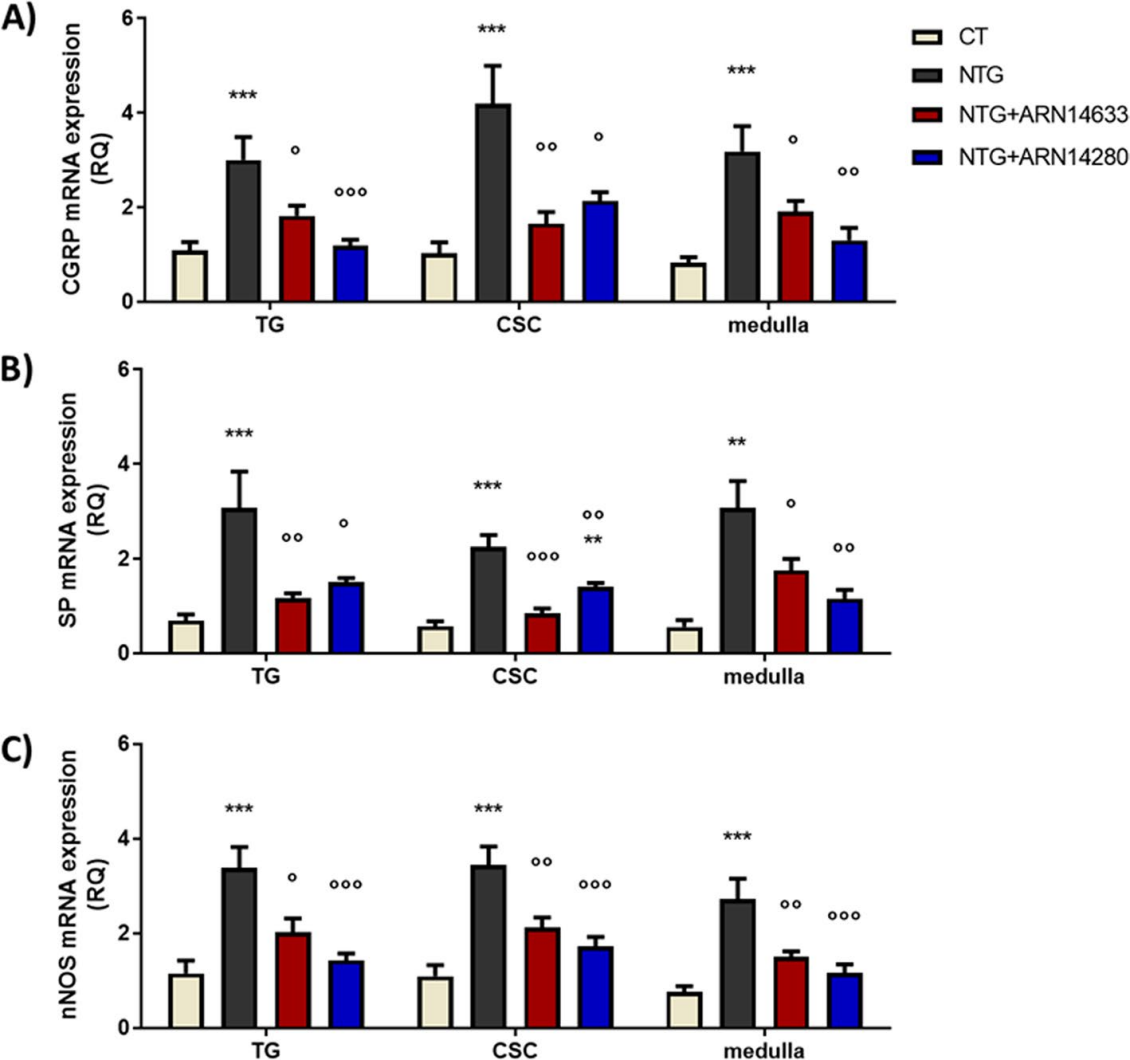
CGRP (**A**), SP (**B**) and nNOS (**C**) mRNA levels (expressed as relative quantification, RQ) in trigeminal ganglion (TG) and cervical spinal cord (CSC) ipsilateral to formalin injection and medulla in toto of animals treated with NTG vehicle (CT), NTG, NTG + ARN14663 and NTG + ARN14280 (n. 9 per group). Data are expressed as mean ± SEM; one-way ANOVA followed by Tukey’s multiple comparisons test; **p < 0.01 and ***p < 0.001 vs CT; °*p* < 0.05, °°*p* < 0.01 and °°°*p* < 0.001 vs NTG

**Fig. 5:**
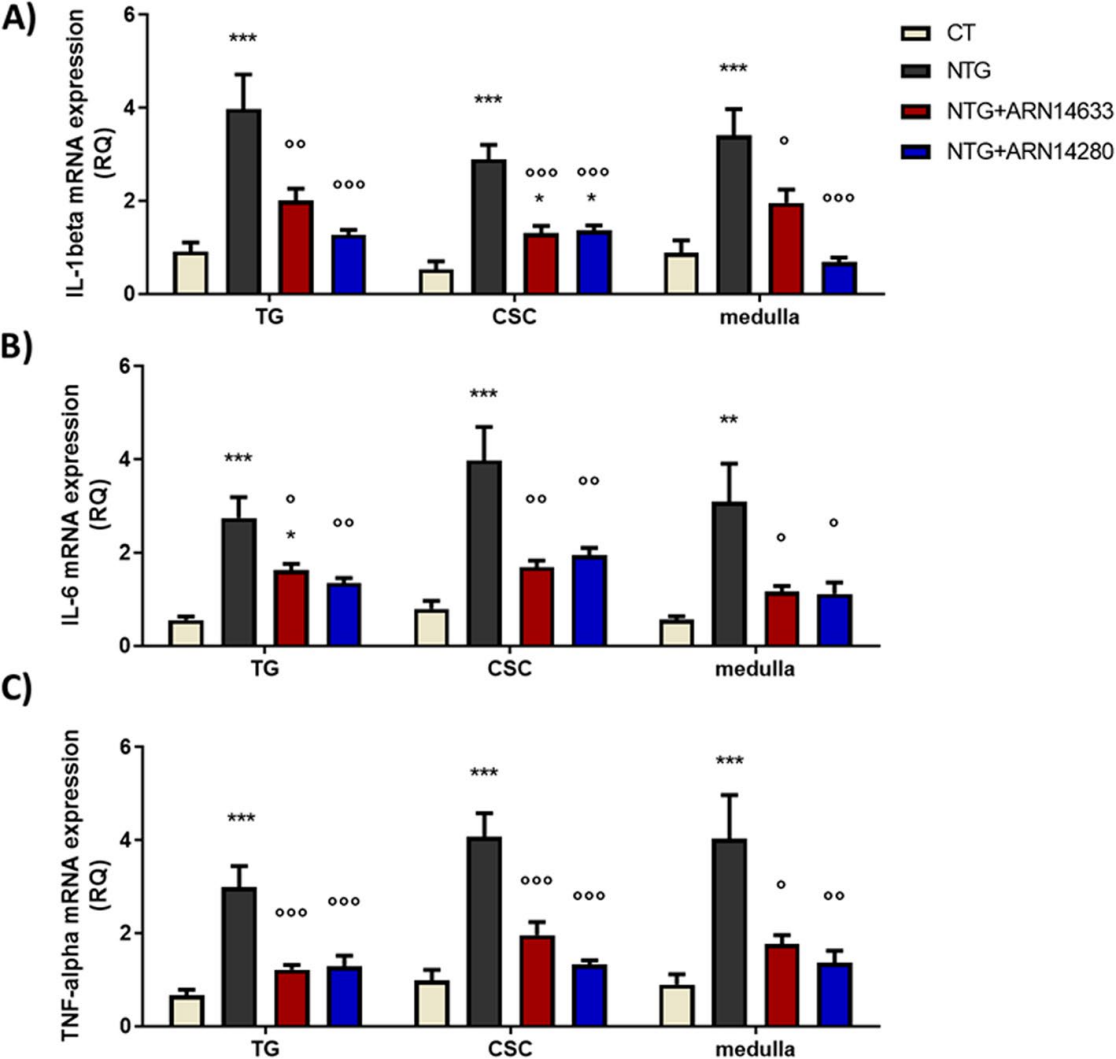
IL-1beta (**A**), IL-6 (**B**) and TNF-alpha (**C**) mRNA levels (expressed as relative quantification, RQ) in trigeminal ganglion (TG) and cervical spinal cord (CSC) ipsilateral to formalin injection and medulla in toto of animals treated with NTG vehicle (CT), NTG, NTG + ARN14663 and NTG + ARN14280 (n. 9 per group). Data are expressed as mean ± SEM; one-way ANOVA followed by Tukey’s multiple comparisons test; *p < 0.05, **p < 0.01 and ***p < 0.001 vs CT; °*p* < 0.05, °°*p* < 0.01 and °°°*p* < 0.001 vs NTG

**Fig. 6:**
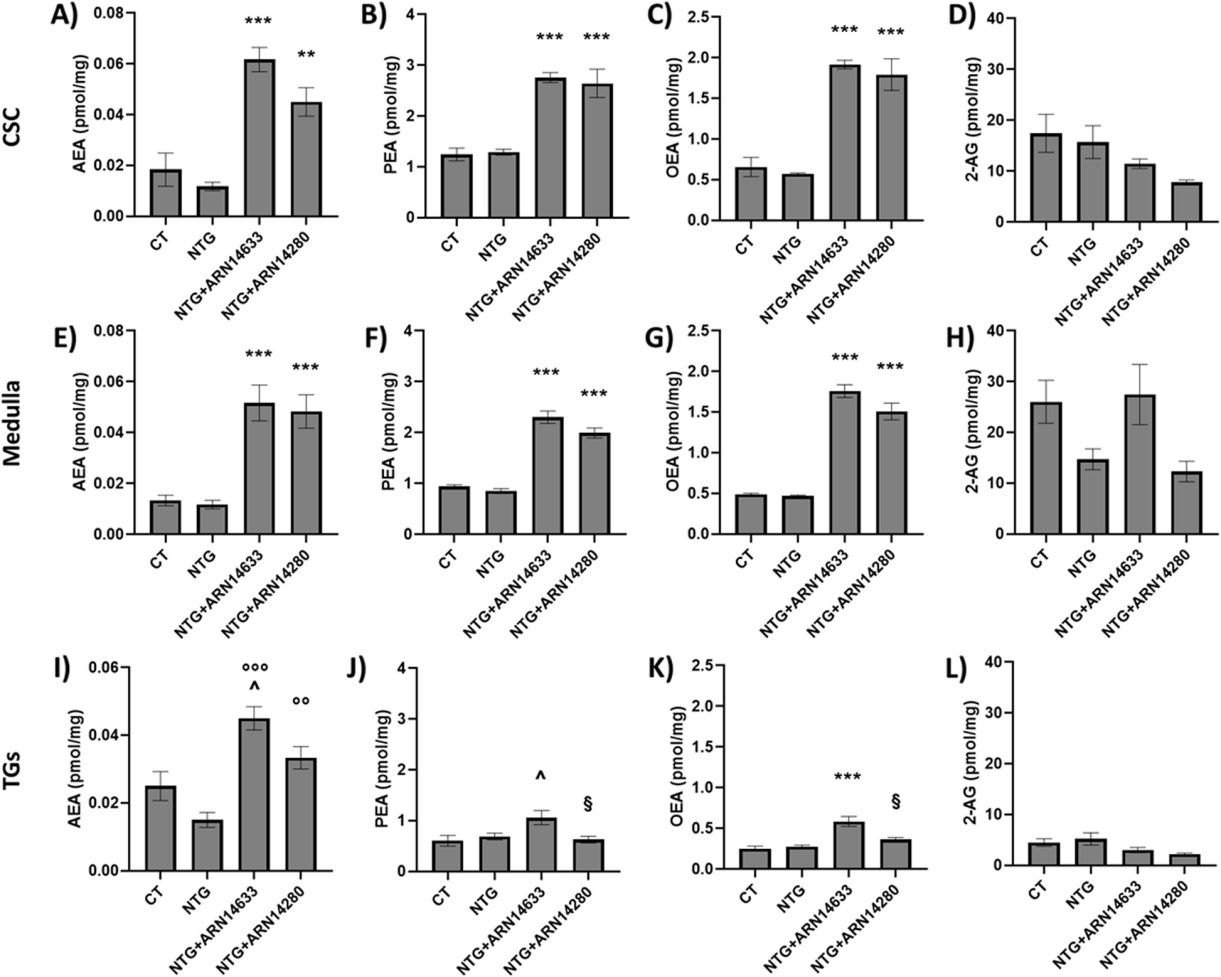
AEA (**A**, **E**, **I**), PEA (**B**, **F**, **J**), OEA (**C**, **G**, **K**) and 2-AG (**D**, **H**, **L**) levels (expressed in pmol/mg) in cervical spinal cord (CSC), medulla and trigeminal ganglia (TGs) of rats treated with NTG vehicle (CT), NTG, NTG + ARN14663 and NTG + ARN14280 (n. 6 per group). Data are expressed as mean ± SEM; Tukey’s multiple comparisons test; **p < 0.01 and ***p < 0.001 vs CT and NTG; °°*p* < 0.01 and °°°*p* < 0.001 vs NTG; ^*p* < 0.05 vs CT; §*p* < 0.05 vs NTG + ARN14633

Treatment with NTG produced a significant increase in the expression of c-Fos, CGRP, SP, nNOS, IL-1beta, IL-6 and TNF-alpha genes in medulla, TG and CSC (Figs. 3, 4 and 5). Administration of either FAAH inhibitor significantly reduced NTG-induced expression of the genes in all areas (Figs. 3, 4 and 5), with the only exception that ARN14280 did not affect c-Fos mRNA levels in the CSC (Fig. 3). ARN14280 and ARN14633 did not influence gene expression when administered to rats that were not pre-treated with NTG (Fig. 4S-6S, supplementary material).

### Endogenous levels of FAAH substrates

**Fig. 7:**
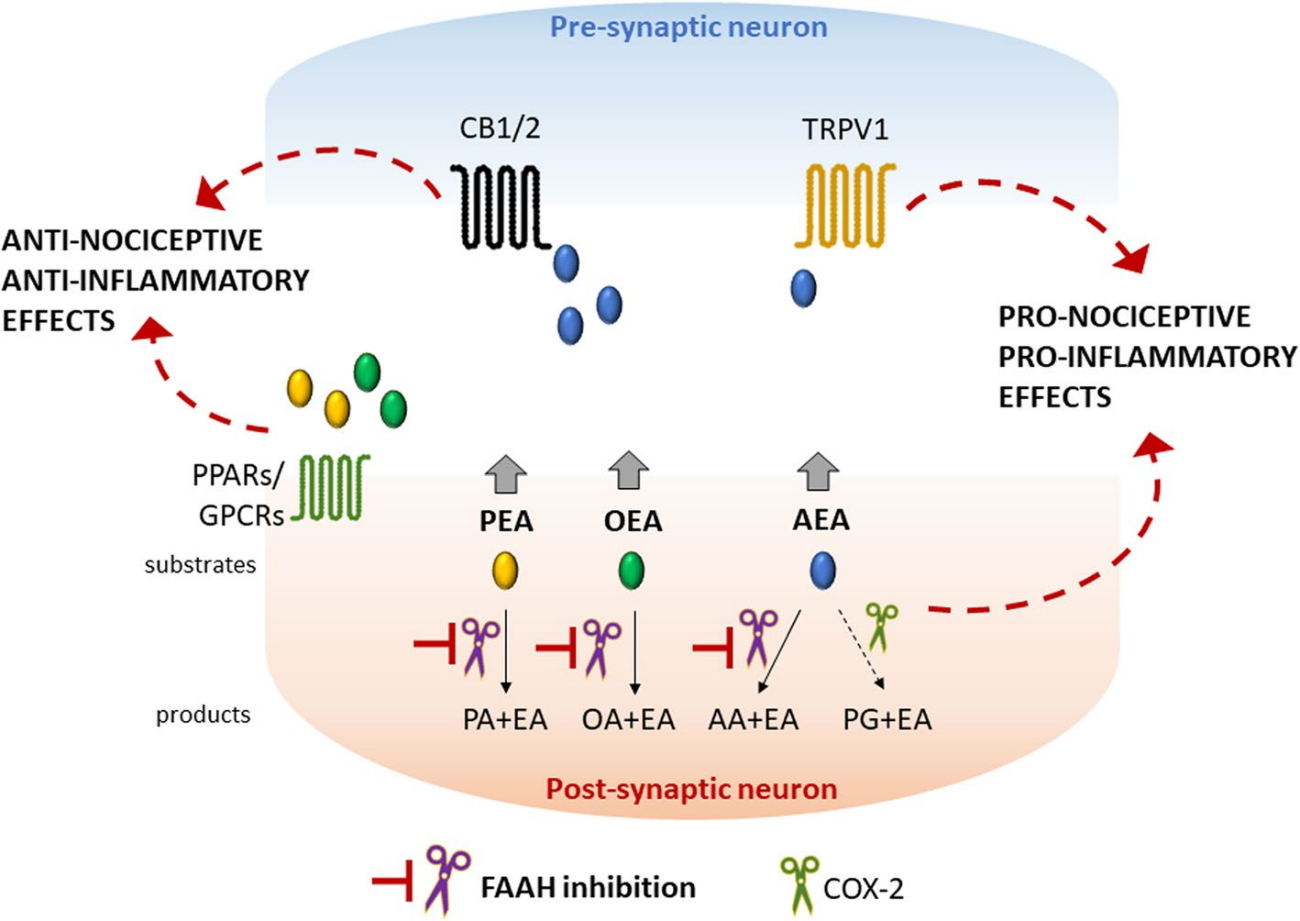
Substrates and products of FAAH with their putative biological functions. The substrates of FAAH enzyme are bioactive amides like AEA, PEA and OEA; for example, AEA is hydrolyzed by FAAH into AA and EA. Inhibition of FAAH leads to an increase of these amides, whose function is to promote anti-nociceptive and anti-inflammatory effects through the activation of CB1/2, PPARs and GPCRs receptors. It must be however noted that, in certain circumstances, AEA can also act on TRPV1 channels causing pro-nociceptive effects. In addition, a small amount of AEA may be metabolized also by COX-2, leading to an increase in pro-inflammatory prostaglandins. For a more comprehensive and detailed description of the endocannabinoids and related lipids metabolism and biological functions, refer to [31, 32, 36]. AA = arachidonic acid; AEA = anandamide; CB1/2 = cannabinoid receptors 1 and 2; COX-2 = cyclooxygenase-2; EA = ethanolamine; FAAH = fatty acid amide hydrolase; Gly = glycerol; GPCRs = G-protein-coupled receptors; OA = oleic acid; OEA = oleoylethanolamide; PA = palmitic acid; PEA = palmitoylethanolamide; PG = prostaglandin; PPARs = peroxisome proliferator-activated receptors; TRPV1 = transient receptor potential cation channel subfamily V member 1

As previously reported [2], NTG treatment did not affect mobilization of FAAH substrates in TG, CSC and medulla compared to the CT group (Fig. 6). Administration of ARN14633 and ARN14280 produced a marked elevation in AEA, PEA and OEA levels in CSC and medulla of animals pre-treated with either NTG or NTG vehicle (Fig. 6 and Fig. 7S, supplementary material); AEA levels were similarly increased in the TGs of animals pre-treated with either NTG or NTG vehicle, while the size of increase in PEA and OEA levels in this area was less marked for ARN4633 and absent for ARN14280 (Fig. 6 and Fig. 7S, supplementary material). By contrast, the treatment with either FAAH inhibitor did not affect 2-AG concentration levels in any of the areas evaluated (Fig. 6 and Fig. 7S, supplementary material).

## Discussion

The biological function of FAAH enzyme is to degrade bioactive amides like AEA, PEA and OEA [4, 31, 32]. FAAH inhibition increases the levels of AEA and related lipids and is associated to analgesia [1, 33–36]. A schematic representation of the putative biological functions of FAAH and its substrates is reported in Fig. 7.

The potential of pharmacological FAAH blockade as a treatment strategy for migraine pain has been the object of prior investigations, which have shown that both globally active (URB597) and peripherally restricted (URB937) FAAH inhibitors are effective in the rat orofacial formalin model of migraine pain [1, 2, 23, 24]. Limitations in analgesic efficacy have been reported with this class of compounds, however. For example, we found that URB597 prevents, but does not reverse, NTG-induced hyperalgesia when administered after NTG [2]. These discrepancies cannot be attributed to a failure to inhibit FAAH activity, as, in all cases, tissue levels of FAAH substrates increased [19, 24]. It is possible, however, that the inability of URB597 to attenuate NTG-induced hyperalgesia might result from pharmacokinetic factors, such as inadequate distribution in relevant biophases. In this report, we examined whether ARN14663 and ARN14280 – two analogs of URB597 with improved solubility and bioavailability – might exhibit greater therapeutic efficacy compared to their parent compound. The present results demonstrate that both ARN14663 and ARN14280 are potent and highly effective at countering NTG-induced trigeminal hyperalgesia in the orofacial formalin test. By contrast, confirming our previous work [2], URB597 had no such effect. Considering that the model utilized here captures several defining features of migraine pain [24, 35], the findings suggest that ARN14633 and ARN14280 interfere with mechanisms that are relevant to this condition and might thus find application in its treatment.

The molecular and cellular mechanisms through which ARN14633 and ARN14280 attenuate hypersensitivity induced by NTG remain to be determined. Our molecular studies do suggest, however, that both agents may at least partly act by modulating neuronal activity in the trigeminovascular system along with transcription of proinflammatory and pronociceptive proteins, effects that are not shared by URB597 [2]. As previously reported in different experimental models [2, 23, 37, 38], we found that both inhibitors reduced c-Fos mRNA levels, a marker of neuronal activation, in central and peripheral areas involved in migraine pain. Reduction of c-Fos transcription was paralleled by a marked reduction in the expression of the genes encoding pronociceptive and proinflammatory peptides (CGRP, SP), proinflammatory cytokines (IL1-beta, IL-6, TNF-alpha), and nNOS. Lending support to the present data, a prior study had shown that the dual FAAH/monoacylglyceride lipase (MGL) inhibitor, which simultaneously blocks the degradation of AEA and 2-AG, lowers serum levels of CGRP in the same animal model of migraine [39].

Discrepancies in the potency and efficacy of FAAH inhibitors with different chemical structures are expected, but often difficult to explain satisfactorily. In addition to inhibitory potency and target residence time, pharmacokinetic factors such as half-life time in plasma and tissue distribution play a major role. In the present study, ARN14633 and ARN14280 were effective at alleviating sensory abnormalities induced by NTG, whereas URB597 was not. By contrast, all three compounds blocked FAAH activity and increased levels of FAAH substrates in the three migraine-related structures under study – medulla, cervical spinal cord, and trigeminal ganglia. This finding is not unprecedented. A prior report showed that systemic administration of URB597 increased levels of FAAH substrates in brain and spinal cord and yet it did not reduce pain behaviors evoked by spinal cord injury [21]. One possible explanation for these incongruities is that ARN14633 and ARN14280 may have access to critical neurovascular structures involved in NTG-induced pain behavior, which are not represented in our tissue survey and are not reached by URB597. Differential biophase access might result, for example, from differences in plasma half-life time, which is much longer for ARN14633 and ARN14280 than URB597 (data not shown). Alternatively, but less likely, both compounds might exert an off-target effect that enhances their efficacy.

## Conclusions

In the last decades, great attention in the research field has been dedicated to the beneficial effects of inhibitors of the endocannabinoids degrading enzymes. Such effectiveness pushed forward their application in humans. For instance, a FAAH inhibitor (JNJ-42165279) has been recently tested in healthy males for the management of anxiety [40]. Moreover, a potent reversible MAGL inhibitor (ABX-1431) has been developed for and tested in other neurological diseases [41]. Our findings confirm the inhibition of FAAH enzyme as a potential therapeutic strategy for migraine pain management and underscore the importance of advancing the chemical and pharmacological optimization of promising lead compounds in association with a careful preliminary evaluation studies in validated animal models. ARN14633 and ARN14280 possess a convincing anti-hyperalgesic profile in a migraine-specific animal model, whose mechanistic basis appear to be linked to a cross-talk of signals that modulate mediators of sensory information, such as neuropeptides, inflammatory cytokines, and nitric oxide [22, 27]. Further studies are needed to elucidate the mechanisms underlying the activity of these compounds, but the present study provides additional information on the role of the endocannabinoid system in pain.

## Supplementary Information

**Additional file 1.** Supplementary materials. Seven supplementary figures for methods and results.

## Data Availability

The data presented in this study are available from the ZENODO repository (doi: 10.5281/zenodo.6497730).

## Abbreviations

2-AG: 2-arachidonylglycerol
AEA: Anandamide
CGRP: Calcitonin gene-related peptide
CB: Cannabinoid
CSC: Cervical spinal cord
CT: Control
FAAH: Fatty acid amide hydrolase
GAPDH: Glyceraldehyde 3-phosphate dehydrogenase
IASP: International Association for the Study of Pain
IL-6: Interleukin 6
IL-1beta: Interleukin 1beta
MGL: Monoacylglyceride lipase
nNOS: Neuronal nitric oxide synthase
NTG: Nitroglycerin
OEA: Oleoylethanolamide
OFT: Orofacial formalin test
PEA: Palmitoylethanolamide
PPAR-α: Peroxisome proliferator-activated receptor- α
SP: Substance P
TG: Trigeminal ganglion
TNF-alpha: Tumour necrosis factor alpha
