# Supplementary material for: Potentiation of endocannabinoids and other lipid amides prevents hyperalgesia and inflammation in a pre-clinical model of migraine
Source: J Headache Pain. 2022 Jul 7;23(1):79. doi: 10.1186/s10194-022-01449-1 (PMC9264488; doi:10.1186/s10194-022-01449-1)
Supplement: Supplementary file 1 — Additional file 1. Supplementary materials. Seven supplementary figures for methods and results. [file 10194_2022_1449_MOESM1_ESM.pptx]

## Slide 1
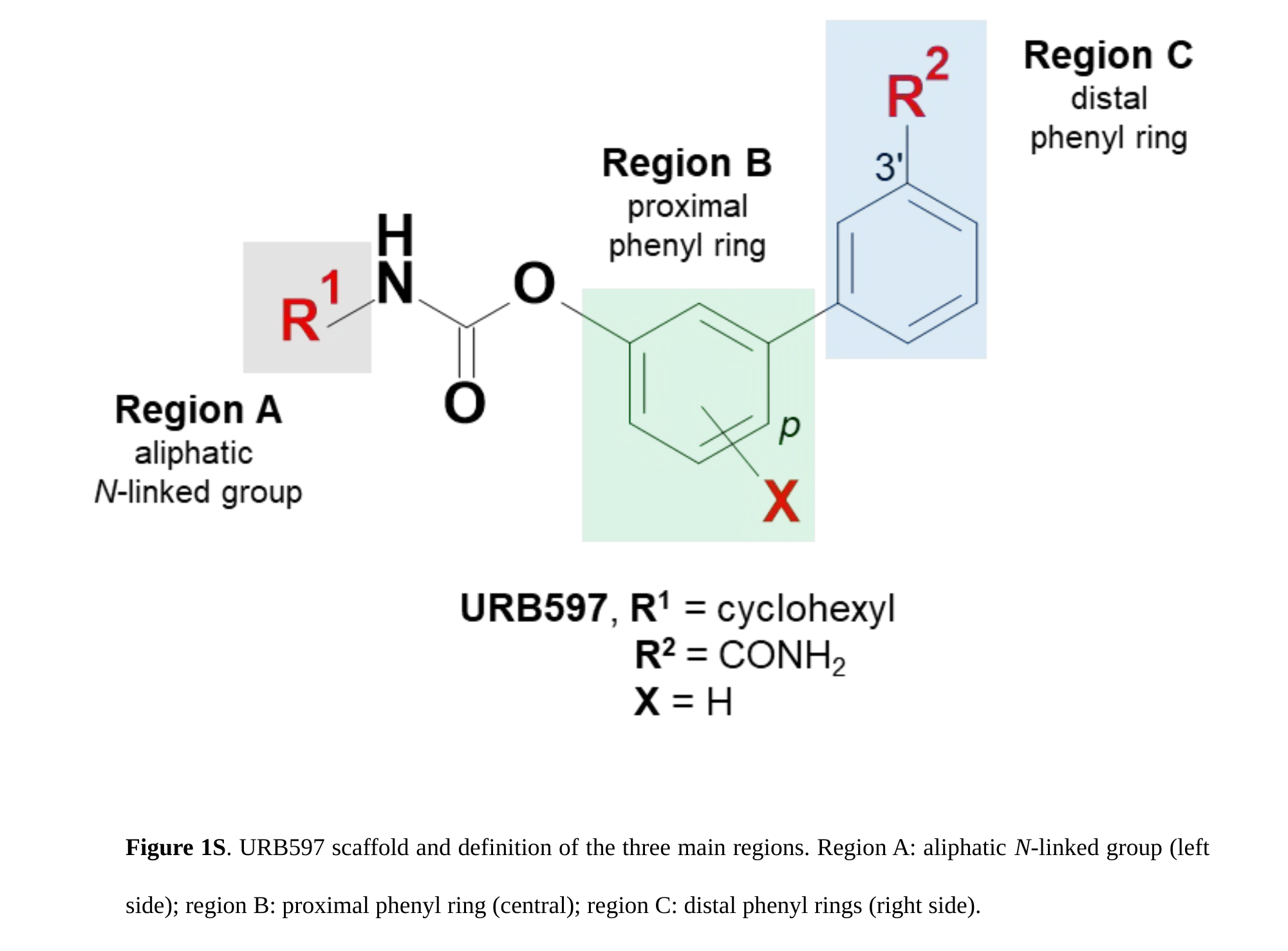

Figure 1S. URB597 scaffold and definition of the three main regions. Region A: aliphatic N-linked group (left side); region B: proximal phenyl ring (central); region C: distal phenyl rings (right side).

## Slide 2
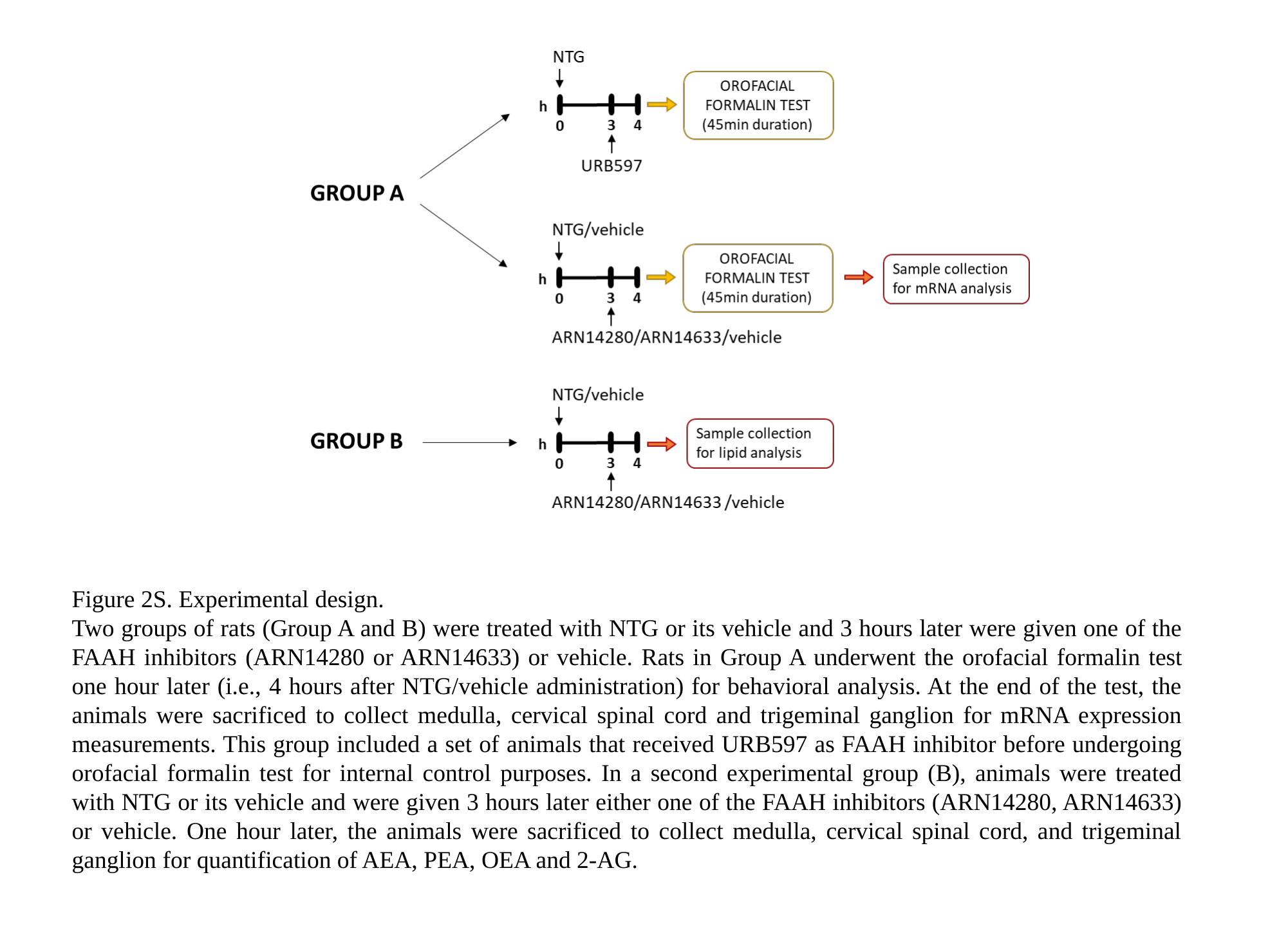

Figure 2S. Experimental design.
Two groups of rats (Group A and B) were treated with NTG or its vehicle and 3 hours later were given one of the FAAH inhibitors (ARN14280 or ARN14633) or vehicle. Rats in Group A underwent the orofacial formalin test one hour later (i.e., 4 hours after NTG/vehicle administration) for behavioral analysis. At the end of the test, the animals were sacrificed to collect medulla, cervical spinal cord and trigeminal ganglion for mRNA expression measurements. This group included a set of animals that received URB597 as FAAH inhibitor before undergoing orofacial formalin test for internal control purposes. In a second experimental group (B), animals were treated with NTG or its vehicle and were given 3 hours later either one of the FAAH inhibitors (ARN14280, ARN14633) or vehicle. One hour later, the animals were sacrificed to collect medulla, cervical spinal cord, and trigeminal ganglion for quantification of AEA, PEA, OEA and 2-AG.

## Slide 3
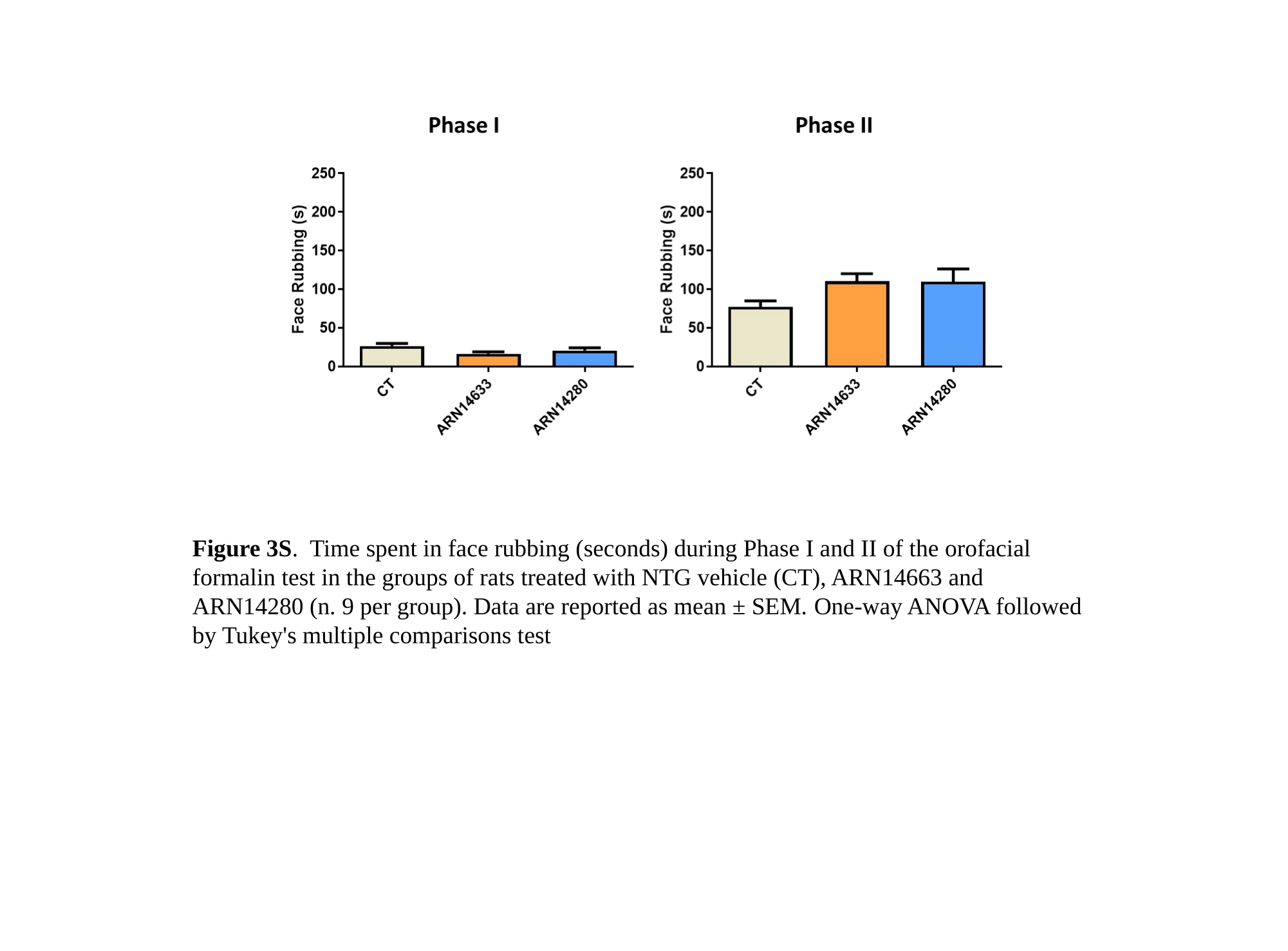

Figure 3S. Time spent in face rubbing (seconds) during Phase I and II of the orofacial formalin test in the groups of rats treated with NTG vehicle (CT), ARN14663 and ARN14280 (n. 9 per group). Data are reported as mean ± SEM. One-way ANOVA followed by Tukey's multiple comparisons test

## Slide 4
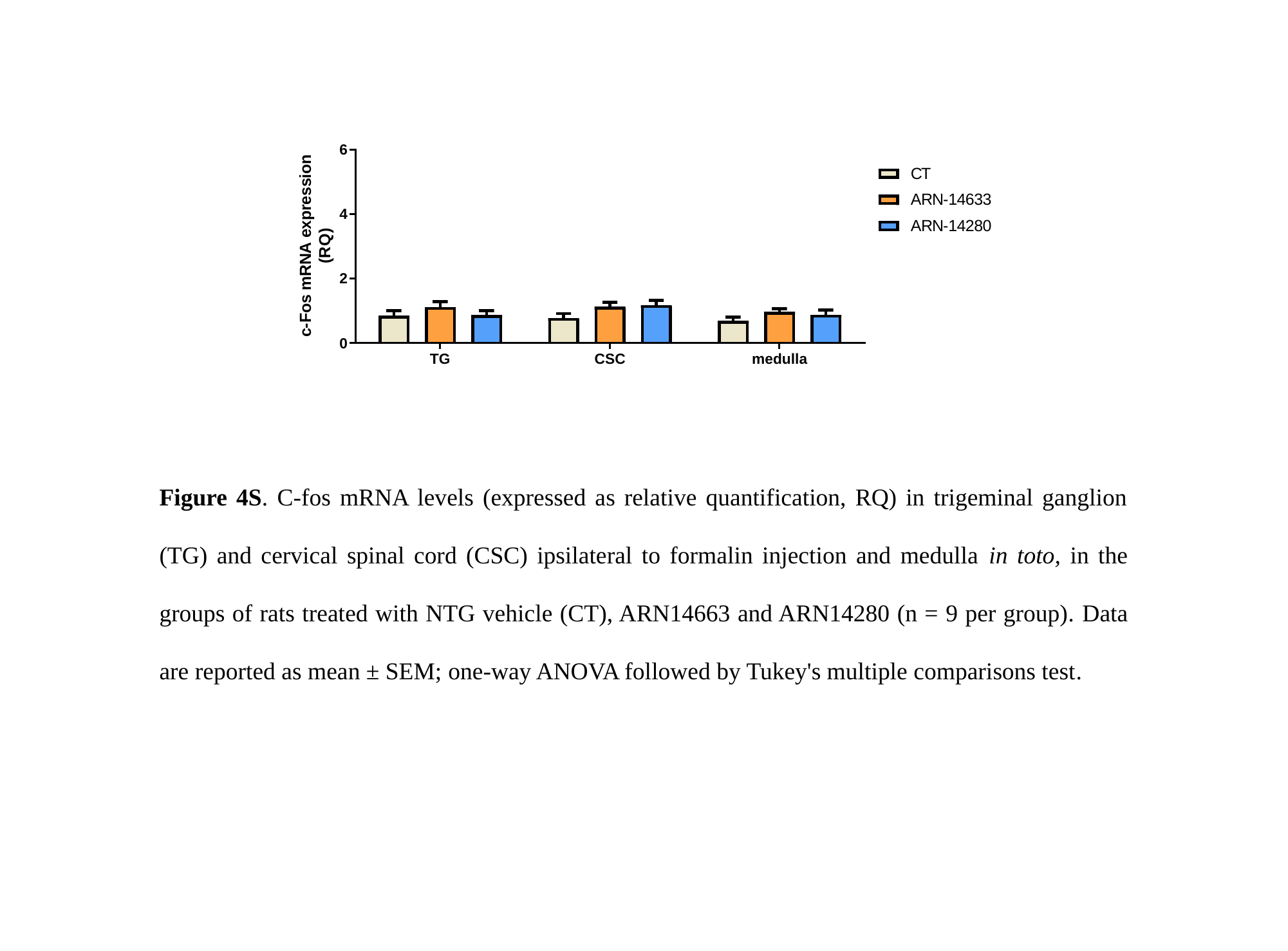

Figure 4S. C-fos mRNA levels (expressed as relative quantification, RQ) in trigeminal ganglion (TG) and cervical spinal cord (CSC) ipsilateral to formalin injection and medulla in toto, in the groups of rats treated with NTG vehicle (CT), ARN14663 and ARN14280 (n = 9 per group). Data are reported as mean ± SEM; one-way ANOVA followed by Tukey's multiple comparisons test.

## Slide 5
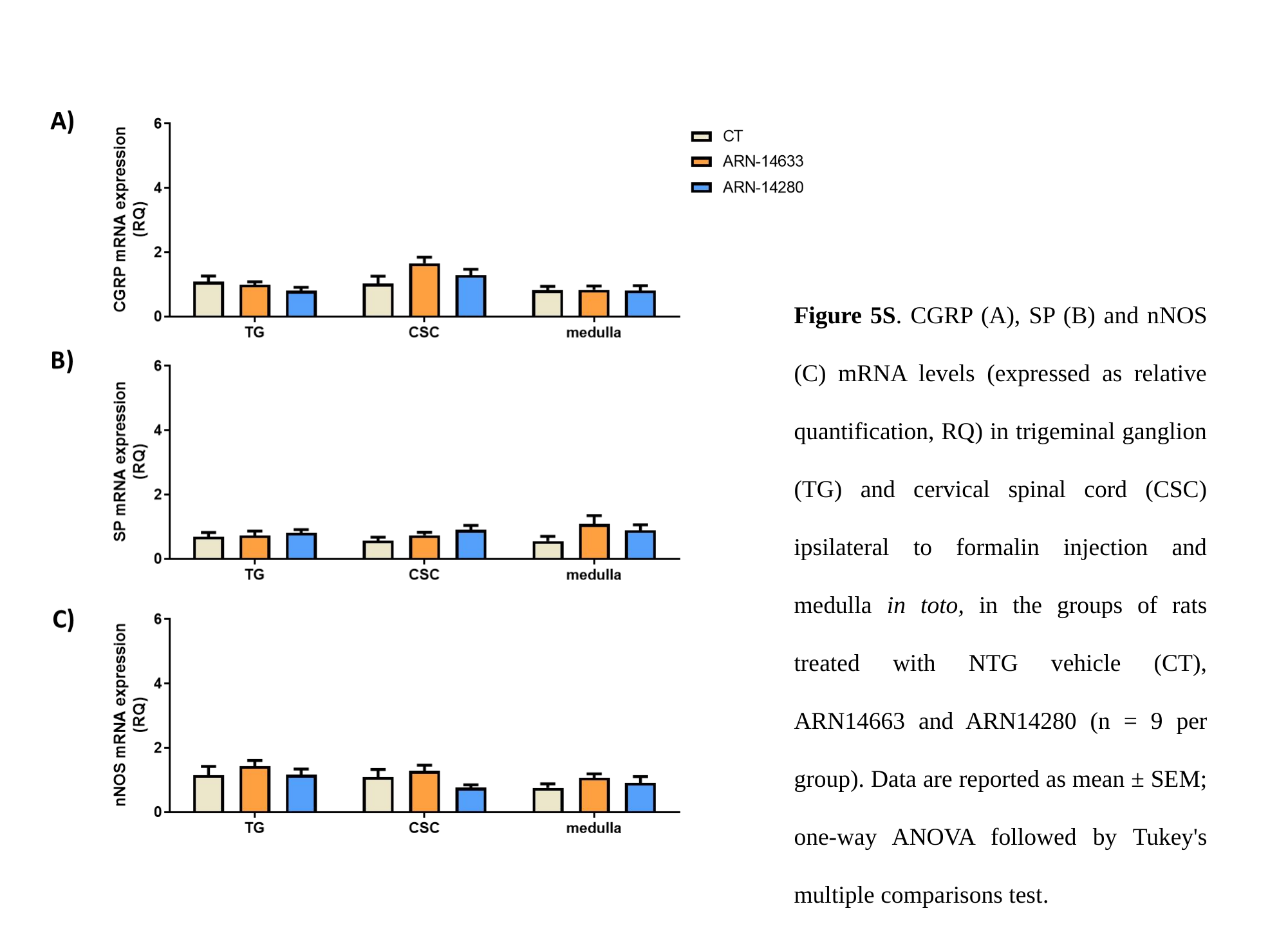

Figure 5S. CGRP (A), SP (B) and nNOS (C) mRNA levels (expressed as relative quantification, RQ) in trigeminal ganglion (TG) and cervical spinal cord (CSC) ipsilateral to formalin injection and medulla in toto, in the groups of rats treated with NTG vehicle (CT), ARN14663 and ARN14280 (n = 9 per group). Data are reported as mean ± SEM; one-way ANOVA followed by Tukey's multiple comparisons test.

## Slide 6
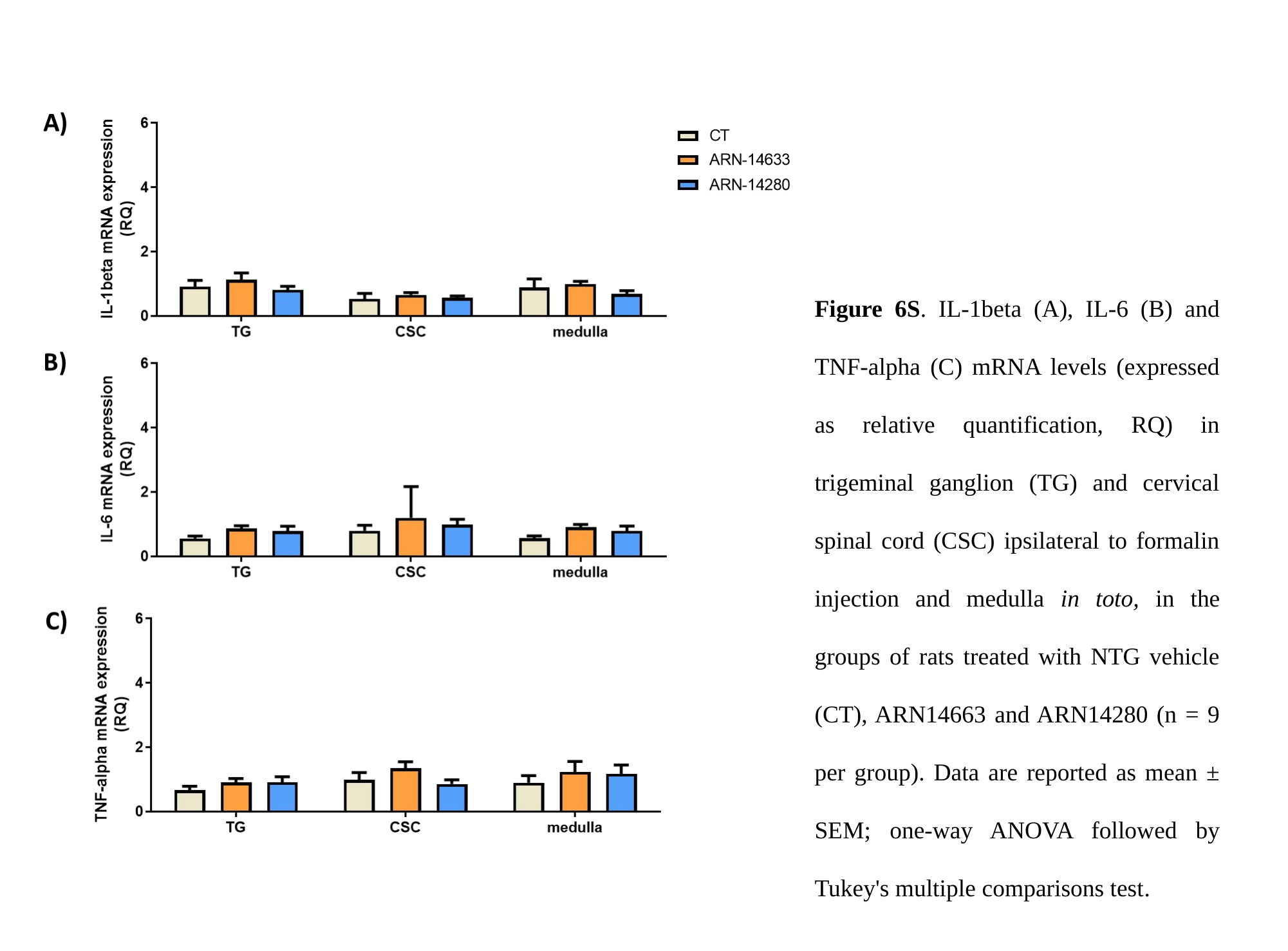

Figure 6S. IL-1beta (A), IL-6 (B) and TNF-alpha (C) mRNA levels (expressed as relative quantification, RQ) in trigeminal ganglion (TG) and cervical spinal cord (CSC) ipsilateral to formalin injection and medulla in toto, in the groups of rats treated with NTG vehicle (CT), ARN14663 and ARN14280 (n = 9 per group). Data are reported as mean ± SEM; one-way ANOVA followed by Tukey's multiple comparisons test.

## Slide 7
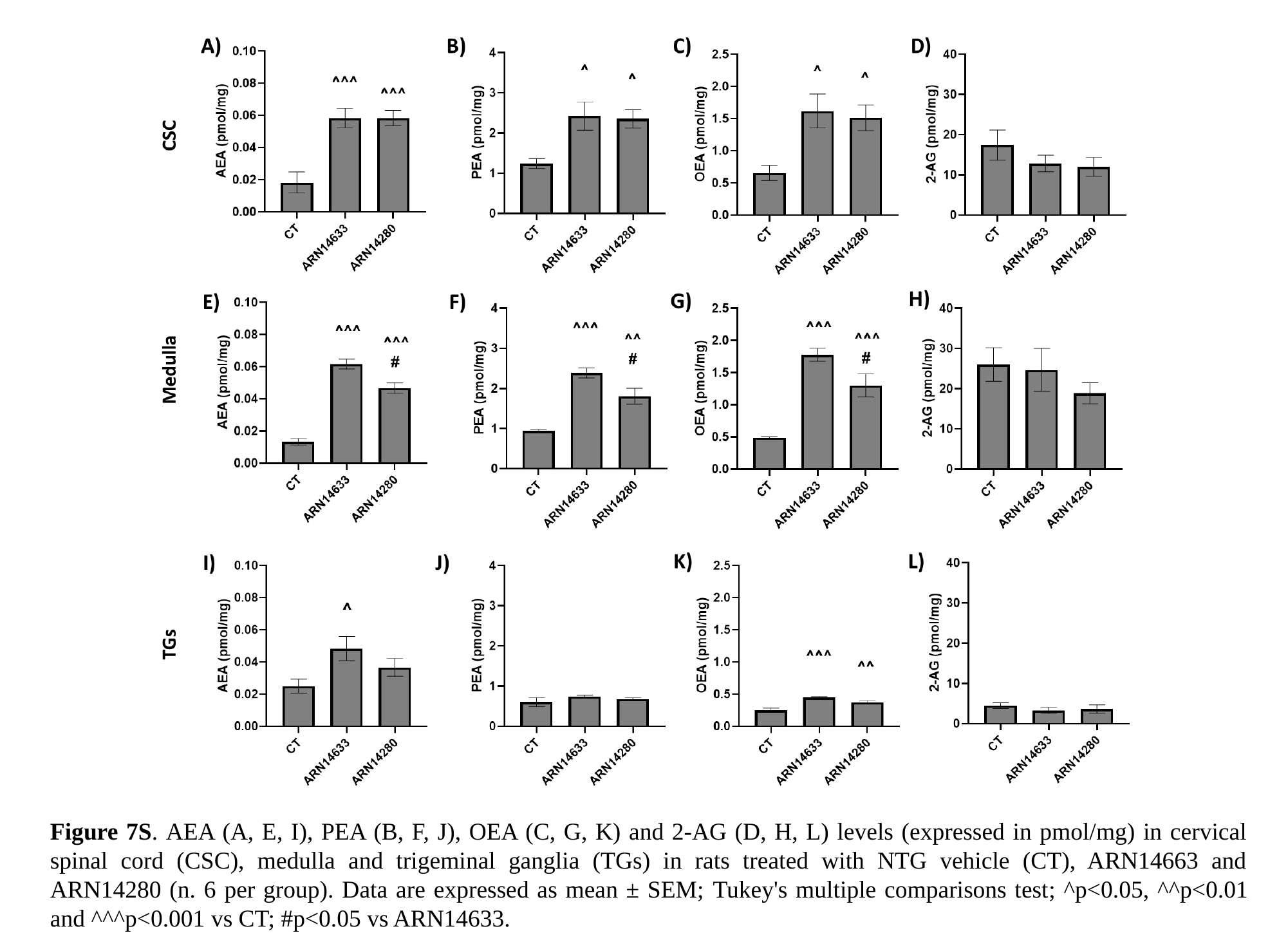

Figure 7S. AEA (A, E, I), PEA (B, F, J), OEA (C, G, K) and 2-AG (D, H, L) levels (expressed in pmol/mg) in cervical spinal cord (CSC), medulla and trigeminal ganglia (TGs) in rats treated with NTG vehicle (CT), ARN14663 and ARN14280 (n. 6 per group). Data are expressed as mean ± SEM; Tukey's multiple comparisons test; ^p<0.05, ^^p<0.01 and ^^^p<0.001 vs CT; #p<0.05 vs ARN14633.
